# Gastric Ulceration in Diabetes Mellitus: Protective Role of Vitamin C

**DOI:** 10.5402/2012/362805

**Published:** 2012-06-16

**Authors:** Daniel U. Owu, Agona O. Obembe, Chukwuemeka R. Nwokocha, Ime E. Edoho, Eme E. Osim

**Affiliations:** ^1^Department of Physiology, College of Medical Sciences, University of Calabar, Calabar, PMB 1115, Nigeria; ^2^Department of Basic Medical Sciences, The University of West Indies, Mona Campus, Kingston 7, Jamaica

## Abstract

The effect of vitamin C administration on gastric acid secretion and ulcer in diabetic rats was studied. Vitamin C (200 mg/kg b.w.) was administered to both streptozotocin-induced diabetic and control groups orally for 28 days. Gastric acid secretion was measured and ulcer was induced using ethanol. Histological changes were observed in the stomach. Basal and stimulated acid secretion in diabetic control rat was significantly (*P* < 0.01) decreased when compared to vitamin C-treated diabetic group and control. Administration of vitamin C significantly (*P* < 0.05) increased the histamine-stimulated gastric acid secretion in diabetics than control while reduction in gastric secretion by ranitidine was similar compared with control. Vitamin C treatment significantly (*P* < 0.05) reduced ulcer index in diabetic group and increased mucus weight when compared with diabetic group which was also confirmed with photomicrographs. The mean body weight of diabetic rats treated with vitamin C was comparable to the control. The blood glucose level was significantly (*P* < 0.01) reduced in diabetic group given vitamin C (8.9 ± 1.8 mMol/L) compared to the diabetic control (32.2 ± 2.1 g). It is concluded that vitamin C is beneficial in improving gastric acid secretion and protects against ulceration in streptozotocin-induced diabetes mellitus in rats due to its antioxidant potential.

## 1. Introduction

Diabetes mellitus is a disease characterised by hyperglycaemia, depletion of antioxidants, and alteration in lipid metabolism [1, 2]. Diabetes is characterised by increased generation of reactive oxygen species and decreased antioxidant levels in the body [3]. It is associated with an increased prevalence of gastrointestinal tract symptoms [4]. Diabetics have increased vulnerability of the gastric mucosa to various ulcerogens such as ethanol, ischemia/reperfusion, stress, and nonsteroidal anti-inflammatory drugs [5–7]. The notable changes that are often observed in patients with chronic diabetes mellitus are decreased gastric secretion (in response to insulin but not to either histamine or pentagastrin) and motility [8–10]. A decrease in gastric acid secretion [11–13] or an increase in acid output with a tendency of developing peptic ulcer [14, 15] in diabetes mellitus has been reported. Other researchers have reported no difference in gastric acid secretion in diabetic compared to nondiabetic conditions [16, 17]. Also acute gastric inflammation and ulcer disease occur with high prevalence in patients with type 2 diabetes mellitus [18]. However, peptic ulcers related to diabetes mellitus are often associated with complications such as gastrointestinal bleeding [19, 20]. 

Vitamin C is one of the antioxidants that exist in the biological system and act as prophylactic and therapeutic agents for various clinical disorders [21]. Vitamin C is the primary water-soluble antioxidant in human plasma that is capable of scavenging oxygen-derived free radicals and sparing other endogenous antioxidants from consumption [22, 23]. 

The beneficial effect of antioxidants and antioxidant enzymes in scavenging free radicals generated during oxidative stress and gastroprotective effect has been reported [24, 25]. These antioxidant systems prevent the generation and actions of reactive oxygen species and provide a potential mechanism for ameliorating diabetic complications. The present study was undertaken to determine whether treatment with vitamin C would restore changes in gastric acid secretion and cytoprotection in stomach from rats with diabetes mellitus.

## 2. Materials and Methods

### 2.1. Drugs and Chemicals

All chemicals were purchased from Sigma Chemical Company (Poole, UK). All drugs were dissolved in distilled water except streptozotocin which was dissolved in citrate buffer.

### 2.2. Experimental Animals

Male Albino rats of Wistar strain weighing between 170–200 g were used for the study after approval by the College Ethical Committee. They were kept under standard laboratory conditions at room temperature of 28 ± 2°C and were exposed to 12 h light/dark cycle. The animals had free access to tap water and standard rat chow. The animals were divided into four groups of animals, namely, normal control (Group 1), control treated with vitamin C group (Group 2), the diabetic control group (Group 3), and diabetic group treated with vitamin C (Group 4). Each group was made up of 12 rats. 

### 2.3. Induction of Diabetes Mellitus

 Diabetes mellitus was induced in two groups of rats by a single intraperitoneal injection of 65 mg/kg body weight streptozotocin (STZ) dissolved in citrate buffer (pH 4.5). Age-matched control rats were injected with the vehicle. Body weight and basal blood glucose levels were measured just prior to STZ injection using animal balance and an automated glucose analyzer (glucometer Acucheck mini plus, Roche, Germany), respectively. A drop of blood sample was collected by a prick on the tail vein. Diabetes mellitus was confirmed 48 h after STZ injection in animals by the presence of blood glucose above 10 mMol/L. The diabetic animals were used for the experiment after four weeks of diabetes mellitus induction.

### 2.4. Administration of Vitamin C

Vitamin C was administered orally as previously described [26]. Briefly, using an orogastric tube, each rat in Groups 2 and 4 was administered vitamin C at a dose of 200 mg/kg body wt. daily for four weeks. Administration was done between 08:00 and 09.00 GMT each day. Rats in the control groups (Groups 1 and 3) received 2 mL of distilled water (placebo).

### 2.5. Measurement of Gastric Acid Secretion

 Gastric acid secretion was measured in all groups of animals by continuous perfusion method of Ghosh and Schild [27] and modified by Osim et al. [28]. Briefly, after an overnight fast, anaesthesia was induced using 25% urethane at a dose of 6 mL/kg body weight intraperitoneally. A cannula was inserted into the trachea to maintain airflow while an oesophageal cannula for infusion of saline was passed through the mouth to the stomach. Another cannula was introduced into the stomach through an incision in the duodenum and was ligated at 0.5 cm from the pylorus. The stomach was firstly flushed using 10 mL saline at room temperature through the oesophageal cannula and then flushed again with normal saline (pH; 7.0) at 37°C at the rate of 1 mL/min using infusion pump (Harvard apparatus, MA, USA). The stomach perfusate was collected every 10 min, and acid output was measured by the titration of the perfusate with 0.01 N NaOH to a pH 7.0 using phenolphthalein as indicator. When a stable acid secretion was obtained, histamine and ranitidine were administered and acid output determined every 10 minutes using the method described above. 

### 2.6. Ethanol-Induced Ulceration

 After 28 days of vitamin C administration, gastric ulceration was induced in the respective animals in another set of experiments. Rats were fasted for 18 h prior to the experiments in mesh-bottomed cages but they had access to drinking water. Ulceration was induced by intragastric instillation of 0.5 mL of 95% ethanol (1 mL/200 g body weight) to rats [29]. One hour later, the rats were anaesthetized with thiopentone sodium (35 mg/kg, i.p.), and the stomachs were removed and opened along the greater curvature and were washed in ice-cold saline and spread on a sheet of cork so as to have a clear macroscopic view of the gastric mucosa. It was examined macroscopically for the presence of ulceration. Ulcer index was calculated based on 0–6-point scale [30]. The scores were as follows: 0 = no lesion,1 = 1–3 small lesions 2 = 1–3 large lesions, 3 = 1–3 thick lesions, 4 = more than 3 small lesions, 5 = more than 3 large lesions, 6 = more than 3 thick lesions. The sum of the total scores was divided by the number of animals to obtain the mean ulcer index for each group. The ulcer scores were performed by two independent examiners who were blinded to the treatment protocol so as to prevent possible experimental bias.

### 2.7. Adherent Mucus

Adherent mucus weight was determined by the method of Tan et al. [31]. Briefly, the mucus covering the stomach wall of both diabetic and control animals was carefully scrapped using a glass slide into a small sample tube containing 1 mL of water whose weight was predetermined. The weight of the container and mucus was taken using a digital electronic balance and the difference taken as the weight of the mucus. 

### 2.8. Histology of the Stomach

Routine histology of the stomach was carried out in parts of the isolated gastric tissues obtained from animal in each group. The tissues were fixed in 10% formalin solution, dehydrated in ascending grades of alcohol, and embedded in paraffin. Sections of 5 *μ*m thickness were taken, stained with hematoxylin and eosin (H & E), and examined by a histopathologist under light microscopy.

### 2.9. Statistical Analysis

The results are expressed as mean ± standard error of mean (SEM). The results were analysed using GraphPad Prism software version 5 (GraphPad Software, San Diego, California, USA). One-way analysis of variance (ANOVA) was used to compare means followed by Bonferroni's multiple comparison test where *P* values were significant. *P* value of 0.05 was considered statistically significant.

## 3. Results

### 3.1. Effect of Vitamin C on Blood Glucose Body Weight and Mucus Secretion

 Diabetic rats showed a significant (*P* < 0.01) increase in blood glucose level and a decrease in body weight as compared to the normal control group. Diabetic rats treated with vitamin C for four weeks showed less hyperglycaemia and a gain in body weight as compared to the diabetic control and vitamin C-treated animals (Table 1). The mucus weight was also significantly (*P* < 0.05) decreased in diabetic group compared with normal control. Vitamin C administration raised the mucus weight in DM group significantly (*P* < 0.05) higher than DM though this was still lower than control. Vitamin C administration had no negative impact on blood glucose level, body weight, and mucus secretion in the rats.

### 3.2. Gastric Acid Secretion 

**Figure 1:**
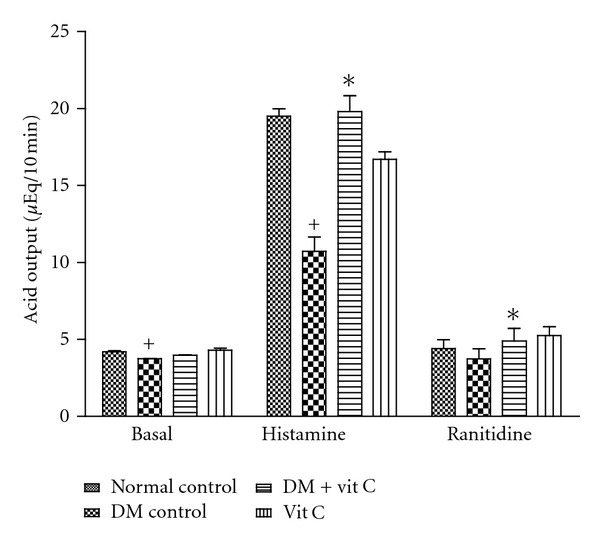
Effect of vitamin C administration on gastric acid secretion in diabetic and control rats. * = *P* < 0.05 compared with diabetic control group. ^+^ = *P* < 0.05 compared with normal control. *n* = 6 in each group.

The basal and histamine-stimulated gastric acid secretion in diabetic rats treated with vitamin C is presented in Figure 1. Basal acid secretion was significantly (*P* < 0.05) reduced in diabetic rats compared with normal control at basal time. Basal acid secretion in diabetic group treated with vitamin C was significantly (*P* < 0.05) reduced though basal acid output in vitamin C supplemented group was significantly (*P* < 0.05) increased when compared with normal control. After 60 min, maximum histamine-stimulated acid secretion was significantly (*P* < 0.05) reduced in diabetic rats (10.72 ± 0.93 *μ*Eq/10 min, *n* = 6) compared with normal control (19.50 ± 0.48 *μ*Eq/10 min, *n* = 6). Stimulated maximum acid output in diabetic group treated with vitamin C was significantly (*P* < 0.05) increased (20.24 ± 1.03 *μ*Eq/10 min) compared with diabetic control. Also, vitamin C supplementation in normal rat also raised stimulated gastric secretion to 16.75 ± 0.51 *μ*Eq/10 min. Administration of ranitidine, an H_2_-histamine receptor antagonist, reduced acid secretion in diabetic control and vitamin C groups to a level comparable to their normal controls, respectively. However, the reduction in diabetic group treated with vitamin C was higher than its normal control (*P* < 0.05).

### 3.3. Gastric Ulcer Study

**Figure 2:**
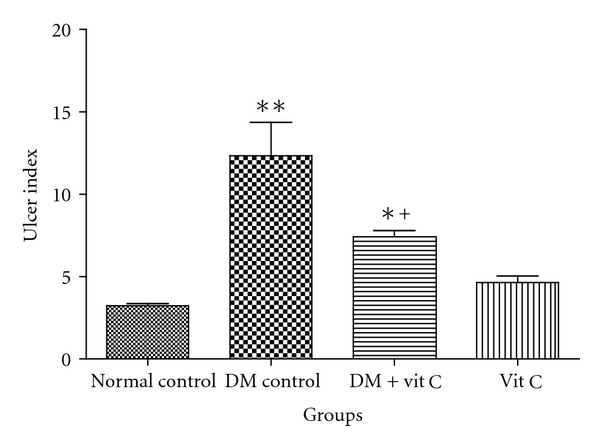
Effect of vitamin C on ethanol-induced ulcer index in diabetic and control rats. ** = *P* < 0.01 compared with normal control, ^+^ = *P* < 0.05compared with diabetic control group, * = *P* < 0.05 compared with normal control group. *n* = 6 in each group.

Diabetic rats showed increased tendency to ulceration in ethanol-induced gastric ulcers model when compared with control. The ulcer index was significantly (*P* < 0.05) higher in diabetic group (12.33 ± 2.04) when compared to normal control (3.23 ± 0.12). Ulcer index in diabetic group administered with vitamin C was 7.42 ± 0.37 while it was 4.6 ± 0.43 in vitamin C group. Vitamin C administration showed significant (*P* < 0.01) ulcer protective activity in diabetic rats when compared with normal control (Figure 2). There was significant (*P* < 0.05) decrease in ulcer index in diabetic group treated with vitamin C when compared with diabetic control. Vitamin C administration, however, showed ulcerogenic tendency in normal rats though this was not significant.

### 3.4. Histological Examination of Gastric Tissue

**Figure 3:**
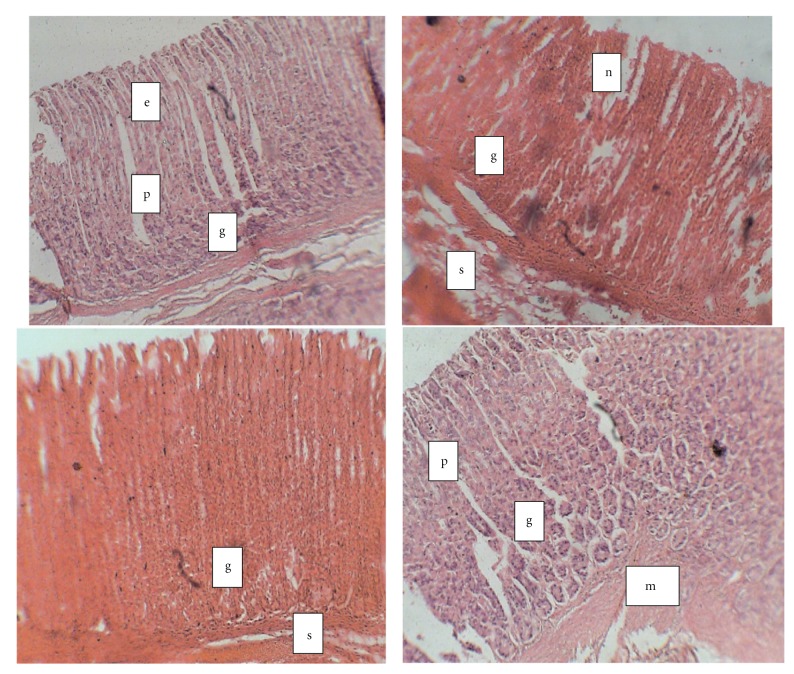
Photomicrograph of gastric tissue from diabetic and control rats treated with vitamin C (H&E; × 100). (a) Normal control; (b) diabetic control; (c) diabetic + vitamin C; (d) Control + vitamin C. e: epithelium; p: gastric pit; g: gastric gland; s: submucosa; mm: muscularis mucosa; n: necrosis.  (a) Stomach from normal control showing: Mucosa is lined by simple columnar epithelium, the gastric pits, underlying gastric glands. The muscularis mucosa and submucosa contain loose areola tissue. (b) Stomach of diabeticcontrol rats showing that mucosa containing tall glandular tissue, the luminal part of which shows some foci of necrosis. (c) Stomach of DM + Vit C showing normal mucosa with tall glandular disposition, no areas of mucosal necrosis. (d) Photomicrograph of the stomach from vitamin C-treated rat showing normal mucosa lined by simple columnar epithelium (e) and numerous gastric glands underneath.

Figure 3 shows light micrographs of the gastric mucosa of normal and diabetic rats treated with vitamin C. Light micrographs of the gastric mucosa of stomach from the diabetic rat showed that the parietal cells were irregularly scattered when compared to normal control. The mucosa showed extensive gastric mucosal lesions and necrosis. The photomicrograph of stomach of diabetic group treated with vitamin C showed normal mucosal outline with tall glandular disposition with no areas of mucosal necrosis.

## 4. Discussion

The present study shows the beneficial effect of vitamin C in protecting the stomach mucosa from peptic ulcer in diabetic rats. It shows that vitamin C provided significant hypoglycaemic and gastroprotective effects in type 1 diabetic rats exposed to acute ethanol-induced gastric ulceration. This study also showed that acid secretion was reduced in diabetic rats when compared to control. This is in agreement with previous reports that in diabetes mellitus, acid secretion in stimulated parietal cells is significantly reduced [13, 32, 33].

The photomicrographs of the gastric mucosa of normal and diabetic rats show that parietal cells are irregularly scattered and show some areas of necrosis in diabetic compared to normal rats. In addition, the epithelial cells appear well defined in the normal but broken and much more scattered in the diabetic rats. This is in line with previous reports that diabetes mellitus has a deleterious influence on the gastrointestinal tract [5, 34]. Lesions of the gastric mucosa in diabetes have previously been reported to include desquamation of the surface epithelium with diffuse hemorrhage and severe hemorrhage with localized erosion [35]. The distortion of the normal morphology of parietal cell in diabetic condition could contribute to the low level of gastric acid secretion. Diabetes induces cellular and functional changes in the glandular stomach especially in the parietal cells such as the decrease in the number of mitochondria accompanied by reduction in H^+^-K^+^-ATPase and canaliculi in parietal cells [36]. This may explain the reduced acid secretion observed in diabetes in our study.

The present study reveals that vitamin C rather offers protection to gastric ulceration and increases gastric acid output in diabetic rats to a level comparable with the control. The possible reason for this could be attributed to its antioxidant property that protects the gastric mucosa against oxidative stress associated with diabetes mellitus [37–39]. Ascorbic acid has been reported to act as a prodrug for formation of ascorbate radical (Asc^•−^) and H_2_O_2_, which can be used therapeutically for treatment of infections [40]. This therefore provided cytoprotection of the mucosa to injury as shown by the increase in adherent mucus on the stomach and a decrease in ulcer index of diabetic rats treated with vitamin C.

The decreased gastric acid secretion in rats could be attributed to gastric mucosal atrophy, possibly as a consequence of diabetic autonomic neuropathy [33]. It could also be due to low concentration of vitamin C, a frequent observation in diabetes mellitus [41–44]. Thus, the administration of vitamin C raised the level in gastric juice and enhanced the acid secretory function in diabetic condition through its cytoprotective role. The histaminergic pathway of gastric acid secretion might have been affected by the diabetic condition as shown by the low acid output on stimulation. The supplementation with vitamin C was shown to increase the stimulated acid output, and the blockage of this pathway by ranitidine seemed not to be affected as there was similar gastric acid reduction in both diabetic and normal conditions. 

Castro et al. [45] had earlier reported that ascorbic acid participates in a general mechanism for concerted glucose transport inhibition and lactate transport stimulation in neuronal and nonneuronal cells. Ascorbate transport into cells occurs by facilitative glucose transporting a member of the GLUT family [46] and Na^+^-dependent, electrogenic process [47]. Our results show that blood glucose level was reduced in diabetic group treated with vitamin C. This is in agreement with a previous study that vitamin C improves whole body metabolism and caused a reduction in blood glucose level in alloxan-induced diabetes mellitus [26]. Thus the inhibition of glucose transport could be a possible mechanism of reduction of blood glucose by vitamin C in diabetic condition.

In summary, the present study has shown that vitamin C administration protects against peptic ulceration in diabetic rats. It also improves gastric acid secretion while protecting the cytoarchitecture of the stomach mucosa. It is concluded that administration of vitamin C stimulates gastric acid secretion and offers protection against ulceration probably owing to its antioxidant property in diabetes mellitus.

## Figures and Tables

**Table 1:** Effect of vitamin C treatment on fasting blood glucose, body weight, and mucus weight in diabetic rats.

Parameter	Normal control	Diabetic control	Diabetic + VIT C	VIT C
Body weight (g)	220 ± 7	184 ± 10^∗^	210 ± 8.7	225 ± 6.8
Blood glucose (mmol/L)	4.2 ± 0.7	>33 ± 2.1^∗^	8.9 ± 1.8^∗†^	4.5 ± 1.1
Mucus weight (mg)	0.97 ± 0.02	0.45 ± 0.02^∗^	0.51 ± 0.02^∗†^	0.88 ± 0.04

^∗^ *P* < 0.05 versus control, ^†^ *P* < 0.05 versus DM, *n*: 6 rats in each group.
